# The Prevention of Senility and a Sanitary Outlook

**Published:** 1907-12

**Authors:** 


**The Prevention of Senility and a Sanitary Outlook.** By Sir JAMES CRICHTON-BROWNE, M.D., LL.D., F.R.S. Pp. 141. London: Macmillan and Co. Limited. 1905.

This little book contains two addresses, one delivered before the Preventive Medicine Section of the London Congress of the Royal Institute of Public Health, the other at a Conference of the Sanitary Inspectors' Association. Both are pleasant reading, and are written in the light, picturesque style characteristic of the author. Without venturing to analyse the factors which together make the charm of this style, one can at once indicate two, namely the gift of humour and the judicious use of good metaphors. As an example of the latter, notice one on page 27. In speaking of the wear and tear of certain occupations, he instances a Sheffield penknife maker, who delivers 28,000 accurate strokes with his hammer daily, each stroke requiring a discharge from the nerve cells of the arm centre. "Little wonder," he says, "that these cells, firing 28,000 rounds a day, should sometimes be overheated and kick, or should generally suffer erosion."

As might be expected, Sir Crichton-Browne has no specific for the prevention or postponement of old age. He does not believe in Metchnikoff's views of the rôle of phagocytes and the bacterial flora of the large intestine in delaying or hastening senility; neither does he think Dr. Allchin's prophecies are sound. "Dr. Allchin," he writes, "has brought forth the *elixir vitæ* from limbo, and tricked her out in the fashionable scientific costume of the hour." Hustling, overeating, insufficient exercise, and luxurious living generally he considers the main enemies of long life.

The second of these lectures, on "The Sanitary Outlook,"

consists of carefully-studied statistics, criticisms on Dr. Maudsley's and Dr. William Hall's views on consumption and the feeding of children, a comparison of the death-rate in urban and rural districts, &c., and the general conclusion that the growth of healthy suburbs by improved means of travelling, and the encouragement of pleasant home life, are the most likely means of improving the future sanitary condition of England.

